# SMRT Sequencing for Parallel Analysis of Multiple Targets and Accurate SNP Phasing

**DOI:** 10.1534/g3.115.023317

**Published:** 2015-10-22

**Authors:** Xiaoge Guo, Kevin Lehner, Karen O’Connell, Jenny Zhang, Sandeep S. Dave, Sue Jinks-Robertson

**Affiliations:** * Graduate Program in Molecular Cancer Biology, Duke University, Durham, North Carolina 27710; † University Program in Genetics and Genomics, Duke University, Durham, North Carolina 27710; ‡ Duke Cancer Institute, Duke University, Durham, North Carolina 27710; § Department of Medicine, Duke University, Durham, North Carolina 27710; ** Department of Molecular Genetics and Microbiology, Duke University, Durham, North Carolina 27710

**Keywords:** PacBio, SNP linkage, barcoded sequencing, rare variant, single-molecule sequencing

## Abstract

Single-molecule real-time (SMRT) sequencing generates much longer reads than other widely used next-generation (next-gen) sequencing methods, but its application to whole genome/exome analysis has been limited. Here, we describe the use of SMRT sequencing coupled with barcoding to simultaneously analyze one or a small number of genomic targets derived from multiple sources. In the budding yeast system, SMRT sequencing was used to analyze strand-exchange intermediates generated during mitotic recombination and to analyze genetic changes in a forward mutation assay. The general barcoding-SMRT approach was then extended to diffuse large B-cell lymphoma primary tumors and cell lines, where detected changes agreed with prior Illumina exome sequencing. A distinct advantage afforded by SMRT sequencing over other next-gen methods is that it immediately provides the linkage relationships between SNPs in the target segment sequenced. The strength of our approach for mutation/recombination studies (as well as linkage identification) derives from its inherent computational simplicity coupled with a lack of reliance on sophisticated statistical analyses.

Next-generation (next-gen) sequencing methods have revolutionized the characterization of entire genomes, epigenomes, and exomes. There remains a need, however, for high-throughput alternatives to traditional Sanger sequencing for repetitive analysis of a single target and for analysis of a small number of targets in multiple samples. In addition, the linkage relationships of single-nucleotide polymorphisms (SNPs) in complex genomes generally requires longer sequence reads than are obtained with common next-gen methods. In the current study, we describe the use of single-molecule real-time (SMRT) sequencing from Pacific Biosciences (PacBio) as a time-efficient, cost-effective, and computationally simple approach that meets these needs.

SMRT technology utilizes a sequencing-by-synthesis approach in which a circular DNA molecule is used as a template for a single DNA polymerase. The first step in the sequencing protocol is conversion of linear amplicons to a circular form by ligation of a DNA polymerase-anchoring, stem-loop adapter (Travers *et al.* 2010) (see Figure 1). The resulting SMRT library is then sequenced using a SMRT cell containing ∼150,000 DNA polymerases anchored in individual nano-chambers. The template-directed incorporation of each nucleotide by a given DNA polymerase releases a unique fluorescent tag that is monitored in real time. The unique strength of SMRT technology relative to other next-gen sequencing methods is single-molecule reads that exceed 10 kb in length. To capitalize on the single-molecule feature and high data output afforded by SMRT sequencing, independent samples can be barcoded during PCR and pooled for SMRT library construction, thereby allowing massive parallel sequencing.

**Figure 1:**
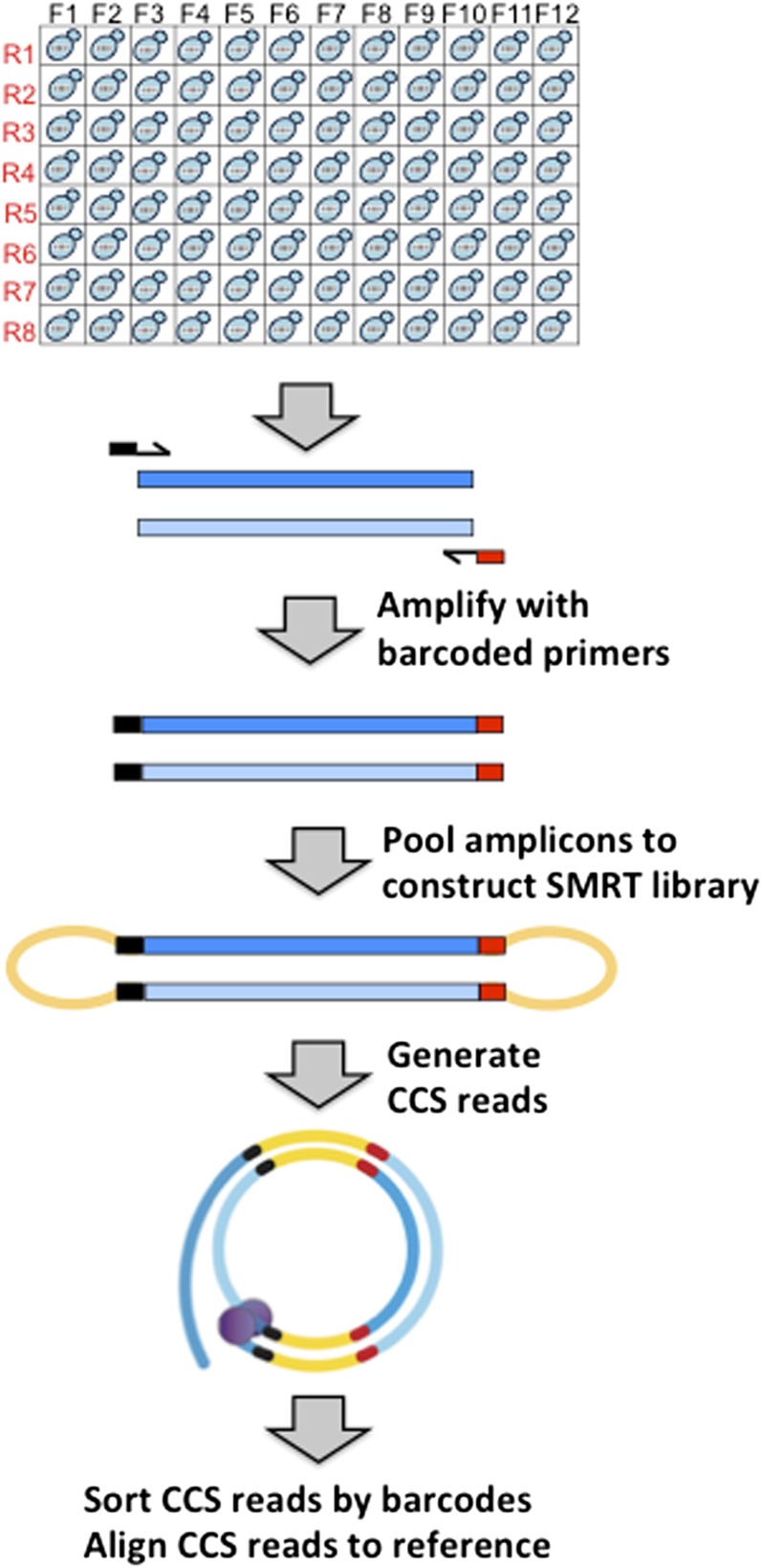
SMRT sequencing pipeline. Each amplicon was barcoded with unique forward and reverse primer pairs in a 96-well format. Complementary amplicon strands are dark and light blue bars; black and red bars correspond to forward and reverse barcodes, respectively, conjugated to target-specific primers (arrows). Following amplicon pooling, hairpin adapters (orange) were attached during SMRT library construction, converting linear into circular molecules. A single DNA polymerase (purple) reads each circular template in real time during the sequencing reaction. Circular consensus sequence (CCS) reads obtained from a SMRT cell were sorted by barcodes and aligned to a reference sequence.

In the current report, we describe development of a barcoding-SMRT pipeline for the repetitive analysis of a target sequence in budding yeast. Following the sorting of individual reads by barcode, sequences are aligned to the appropriate reference for analysis. The utility of this approach in the molecular analysis of recombination intermediates and in the rapid construction of mutation spectra is demonstrated. The high coverage obtained through repetitively sequencing a single mutation target additionally allowed determination of the error frequency at each position within a 2-kb amplicon. These error frequencies are relevant to identification of rare variants in more complex samples. Finally, the generality and robustness of the barcoding-SMRT approach for mutation identification and SNP phasing in a complex genome is demonstrated through analysis of target amplicons from primary lymphomas and derived cell lines.

## Materials and Methods

### Yeast strains and growth conditions

Low- and high-transcription strains (*pCAN-CAN1* and *pGAL-CAN1*, respectively) were previously described (Lippert *et al.* 2011). SJR3956 was used to identify recombinants after galactose induction of an I-*Sce*I generated double-strand break and was derived from W303 (*MATa RAD5 leu2-3,112 ura3 CAN1 his3-11,15 ade2-1*). Briefly, the strain contained a *lys2* allele cleaved by I-*Sce*I (chromosome II) and a 4-kb repair template with evenly spaced SNPs (chromosome V). Repair of the broken molecule via homologous recombination generated a selectable Lys^+^ phenotype.

Cells were grown nonselectively in YEP (1% Bacto-yeast extract, 2% Bacto peptone, 500 µg/ml adenine hemisulfate) supplemented with 2% glycerol and 2% ethanol for mutation analysis or with 2% raffinose for recombination experiments. Canavanine-resistant (Can-R) mutants were selected on synthetic complete glucose (SC) medium lacking arginine and supplemented with 60 μg/ml canavanine. For identification of recombinants, I-*Sce*I was induced by 0.1% galactose for 40 min and Lys^+^ recombinants were selected by plating on SC-lysine medium. All growth was at 30°.

### Yeast sample preparation and SMRT sequencing

Barcoded primers contained 20 nt of yeast-specific sequence conjugated to 16 nt of PacBio-designated forward and reverse barcodes F1–F24 and R1–R16, respectively (Supporting Information, Table S1). Specific barcode pairings used were F1–F12 with R1–R8, F1–F12 with R9–R16, F13–F24 with R1–R8, and F13–F24 with R9–R16. Custom oligonucleotides were synthesized by Eurofins MWG Operon and were provided in a 96-well format. To amplify *CAN1* when under control of its endogenous promoter, forward and reverse primers contained yeast-specific sequences 5′- CCCTCCGAAGGAAGACTCTC and 5′-GGGAGCAAGATTGTTGTGGT, respectively. To amplify *pGAL-CAN1*, the forward primer above was replaced with primer 5′-AATCTGTCGTCAATCGAAAG. For analysis of recombinants, the repaired allele was amplified using yeast-specific sequences 5′-GAGTGTATGGGCTGCATTGA and 5′-TTGGGAGTTGGGAATTGAAG conjugated to forward and reverse barcodes, respectively.

Genomic DNA was isolated from unpurified yeast mutants/recombinants grown nonselectively for 3 d in 96-well microtiter plates. *CAN1* or *LYS2* sequences were then amplified using *Taq* polymerase (Bioline) and barcoded primers in the same 96-well format. Ten microliters of barcoded product from each mutant/recombinant were pooled, and the pooled sample was purified using the GeneJet PCR Purification Kit (Thermo Scientific). Six micrograms were sent to the Duke Center for Genomic and Computational Biology Core, where SMRTbell adapters were ligated to the barcoded amplicons (Travers *et al.* 2010). Sequencing of the resulting library was performed on a PacBio RSII instrument using P6-C4 chemistry and movies of 180 min.

### Human DNA preparation and SMRT sequencing

DNA was extracted from diffuse large B-cell lymphomas (DLBCLs) or derived cell lines using the Qiagen Allprep RNA/DNA/Protein kit (Qiagen 80004). Briefly, 10^6^ cells were centrifuged at 1100 rpm for 5 min, washed in PBS, and lysed in 600 μl RLT buffer. The lysate was passed through an AllPrep DNA spin column, which bound genomic DNA. The column was washed in AW1 buffer followed by a wash in AW2 buffer; DNA was eluted in 100 μl of TE buffer and quantified using a NanoDrop; 25 ng genomic DNA were used in individual PCR reactions and the following human sequences were contained within barcoded forward and reverse primers, respectively: 5′-GTAGAGGTGGGCACCCCCGCCTCCGCACTT and 5′-TGTGGCAACTTAGTTCAGCATATCCCCTGG for MYD88 exons 1-4, 5′-GGTAAGCTCAACCCTGCTCTGGCAAGAGAA and 5′-TGCATGGCAGCTAAATGCCTCAACAAGATC for MYD88 exon 5, and 5′-CCCCCCAAACAACTTACCATTGTAC and 5′-GTTCTCTTTCCCATTCGGTCTGCGC for EZH2 exon 16.

### SMRT sequence analysis pipeline

Data were analyzed using an in-house pipeline (SmrtSeqTool) written in Python that uses Bowtie2 for read alignment and SAMtools/Bcftools for variant calling (Shander *et al.* 2009). The pipeline (https://sourceforge.net/projects/smrtseqtool/) was run on a standard Mac with OS X Yosemite. Files needed to run the SmrtSeqTool analysis pipeline are: (1) a FASTQ file containing the PacBio-generated CCS reads (“.fastq” extension); (2) reference sequence (ref.txt) (Gibson *et al.* 2010); (3) forward barcodes (ForBar.txt); and (4) reverse barcodes (RevBar.txt). Templates for files 2–4 are available in the “Sample” folder that can be downloaded from the website. The *CAN1* and *LYS2* reference sequences were from the *Saccharomyces* Genome Database (SGD); the MYD88 (Genbank, MYD88) and EZH2 (Genbank, EZH2) reference sequences were from GenBank.

The first step of the pipeline uses the forward and reverse barcodes to generate different forward and reverse barcode pairs. Additionally, all 12 consecutive nucleotide windows for each barcode pair are generated. The CCS reads in FASTQ format are then sorted according to these barcode pairs. Each CCS read is classified according to a forward barcode and a reverse barcode. For each unique barcode pair, we demand that at least 12 of 16 nucleotides of the sequenced molecule match each barcode. CCS reads matching this criterion are written into the corresponding barcode files named F1R1, F1R2, etc. In our runs, none of the reads was assigned to more than one barcode combination. Once CCS reads are sorted, the pipeline uses Bowtie2’s default alignment (end-to-end read alignment) to align reads to the corresponding reference sequence and outputs a set of alignments in SAM format. The SAM alignment files are then processed and analyzed by SAMtools, followed by variant calling using its built-in Bcftools. For the purpose of our data analysis, SAMtools default parameters were used. Finally, each individual CCS read alignment was visualized using the Text Alignment View (tview) in SAMtools.

## Results

A barcoding-SMRT sequencing pipeline was developed using budding yeast and applied to the analysis of strand-exchange intermediates generated during recombination and to the analysis of mutation spectra. To allow parallel sequencing of hundreds of independent mutants/recombinants, a 2-kb amplicon from each was tagged with a unique forward–reverse barcode combination and the tagged amplicons were pooled to construct a single SMRT library. Although SMRT sequencing has only ∼90% accuracy during a single read of a given template, a DNA polymerase can traverse a circular molecule multiple times before stochastically disengaging. This property allows construction of a circular consensus sequence (CCS) read that minimizes the contributions of random errors. We set a parameter of at least three complete passes of a given molecule for building a CCS read (*i.e.*, at least two reads of one strand and one read of the complementary strand) as the default setting of two produced poor-quality sequences. Following the sorting of CCS reads by forward–reverse barcode pairs, sorted reads were aligned back to the reference sequence using Bowtie2. The data reported here were generated using PacBio P6-C4 chemistry; the average read length was ∼15 kb, and ∼55,000 CCS reads of 2-kb amplicons were obtained per SMRT cell. The pipeline for our analysis is available at https://sourceforge.net/projects/smrtseqtool/.

### Barcoding-coupled SMRT sequencing for analysis of recombination intermediates

We have developed recombination assays that monitor the strand-exchange intermediates formed during repair of a defined double-strand break (DSB) in yeast (Mitchel *et al.* 2010). The key features of these systems are: (1) use of a diverged sequence with evenly spaced single-nucleotide polymorphisms (SNPs) as a repair template for a broken molecule and (2) use of mismatch-repair defective background, which prevents repair of mismatches formed during strand-exchange reactions. In early studies, substrates were small enough to be sequenced in a single Sanger reaction and mismatches created by strand exchange during recombination were detected manually as dual peaks on sequencing chromatograms (Mitchel *et al.* 2010; Guo and Jinks-Robertson 2013). Given the labor-intensive nature of the analysis, especially with substrates that exceed ∼800 bp, a SMRT sequencing approach was developed. This was coupled with amplicon barcoding to allow the simultaneous sequencing of multiple recombinants.

**Figure 2:**
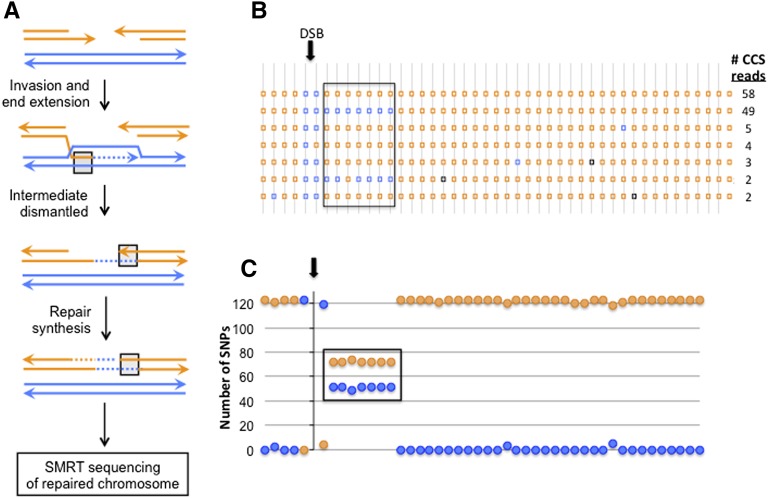
Mapping strand exchange intermediates during recombination. (A) Model of double-strand break (DSB) repair that generates mismatched SNPs (blue paired with orange strand) on only one side of the initiating break. (B) SNP linkages in 123 CCS reads. SNPs are spaced at ∼50-bp intervals and are indicated by orange or blue squares. Isolated blue or black squares within orange regions represent random errors that matched the blue SNP or did not match either SNP, respectively. (C) Sum of orange/blue SNPs (circles) at each position. In all panels, the region of strand exchange where orange and blue strands pair is boxed.

Figure 2A illustrates a mechanism of DSB repair that generates mismatches on only one side of the initiating break; blue and orange lines reflect the diverged recombination substrates. The 123 CCS reads obtained for a specific recombinant of this type, which was contained within a SMRT library of 96 recombinants, are presented in Figure 2B. The number of single-molecule reads in each class is indicated, and the data are summarized in Figure 2C. Within the repaired orange molecule, there is a contiguous region where 51 of 123 single-molecule reads had blue SNPs (each square/circle is a SNP) instead of orange, revealing precisely the extent and position of strand exchange (boxed) during repair of the DSB. This specific application of SMRT sequencing demonstrated the general utility of the barcoding approach, the highly accurate phasing of SNPs within an amplicon, and a low error frequency (black squares in Figure 2B) associated with sorted CCS reads. Below, we extend the general framework to mutation analysis in yeast and to human tumor samples.

### Biological validation of the barcoding-SMRT approach for analysis of mutation spectra

Characterization of mutation signatures and patterns reveals underlying DNA sequence properties that promote instability, genetic consequences of endogenous and exogenous mutagens, and the substrates of enzymatic machineries that remove premutagenic DNA lesions. Forward mutation assays provide an unbiased view of all mutations types, but the target size often exceeds the limits of a Sanger-sequencing reaction. The yeast *CAN1* forward mutation assay was used to demonstrate feasibility of SMRT technology for accurate, parallel sequencing of hundreds of mutants. The *CAN1* locus has a 1.7-kb open reading frame and encodes arginine permease. The wild-type (WT) protein transports the toxic arginine analog canavanine into cells, resulting in death. Cells with a defective gene are resistant to canavanine (Can-R) and form colonies on canavanine-containing medium. Following the isolation of DNA from independent Can-R mutants, a 2-kb fragment from each was amplified using primers tagged with unique forward–reverse barcode combinations.

**Figure 3:**
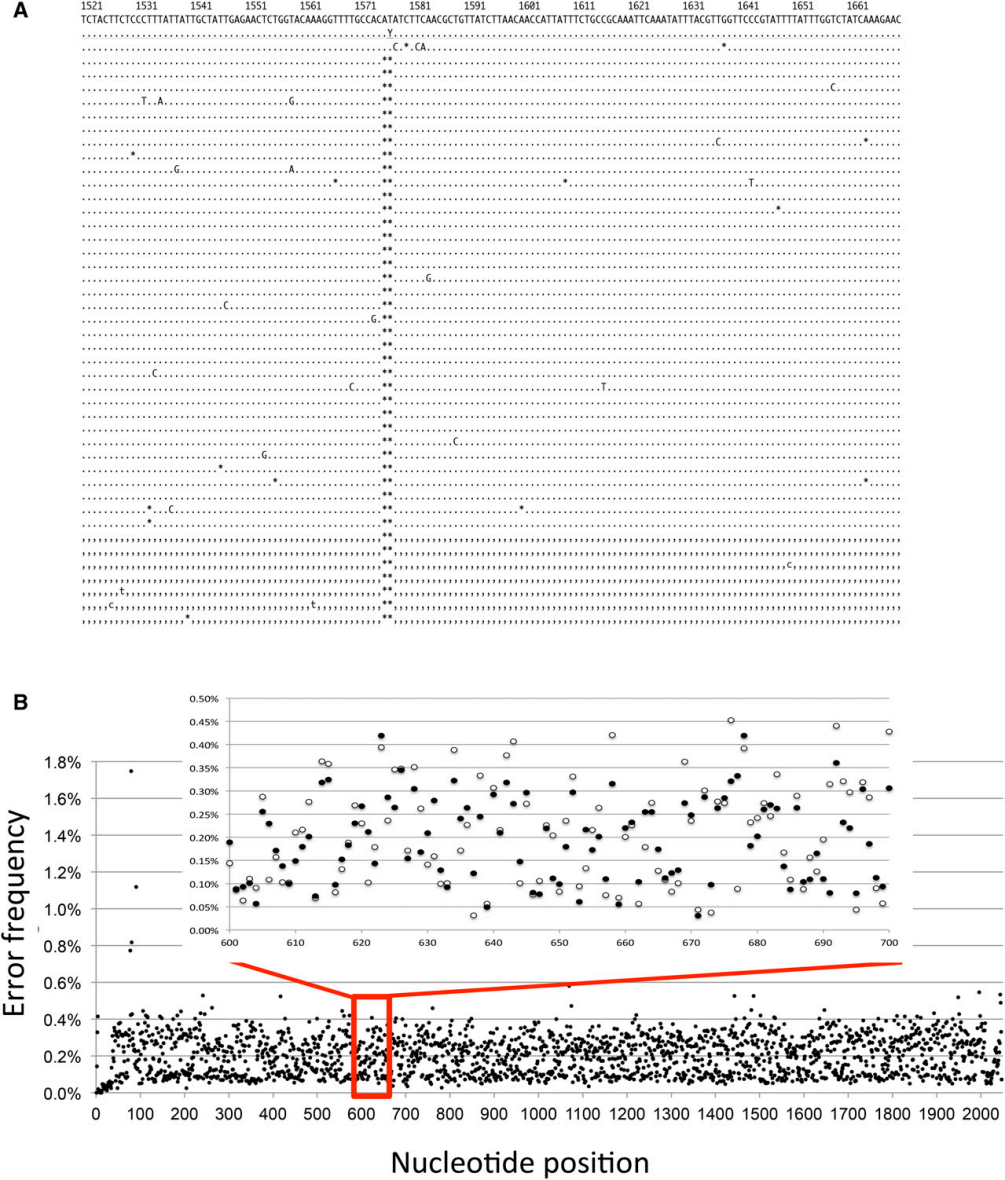
*CAN1* sequencing data. (A) Alignment of CCS reads for a Can-R mutant harboring a 2-bp deletion. Dots and commas are matches to complementary strands; letters are deviations from the reference sequence and asterisks are deletions. (B) Base-substitution error frequency at each position in the *CAN1* amplicon. The enlarged inset compares error frequencies derived from two independent SMRT libraries indicated by open and filled circles. See also Figure S1.

Validation that SMRT sequencing captures known biological profiles was obtained by sequencing Can-R mutants isolated under normal transcription conditions or when transcription was highly elevated. We and others have demonstrated that, under high-transcription conditions, forward mutations in *CAN1* are dominated by short, 2- to 5-bp deletions rather than by base substitutions (Lippert *et al.* 2011; Takahashi *et al.* 2011). Of 141 Can-R mutants isolated under low-transcription conditions, 92% contained a single base substitution, 7% had a 1-bp insertion/deletion (indel), and 1% had complex changes. This is similar to the mutation distribution reported by others using Sanger sequencing (Lang and Murray 2008; Lippert *et al.* 2011; Guillet *et al.* 2006). When Can-R mutants were isolated under high-transcription conditions, however, 2- to 5-bp deletions were the causative mutation in ∼61% (85/139) of the mutants sequenced. An example of aligned CCS reads derived from a mutant with a 2-bp deletion is shown in Figure 3A. Within the set of high-transcription mutants, we also detected a large, 40-bp internal deletion as well as complex sequence changes (examples are shown in Figure S1). In principle, there is no limit to the size of deletion or insertion that can be detected by SMRT sequencing, as long as the primer binding sites remain and amplicon production remains efficient. Although the average coverage of each mutant usually exceeded 100, the high accuracy of SMRT sequencing suggests that a causative mutation can be identified from only 5 to 10 CCS reads. To date, we have combined a maximum of ∼400 independent mutants in a single SMRT library, which was then sequenced in two SMRT cells. An average coverage per mutant of ∼100 CCS reads was obtained, indicating the feasibility of sequencing several thousand barcoded mutants in a single SMRT cell.

### Identification of rare variants in a complex sample

SMRT sequencing has a single-pass error rate of 10–15%, with >90% of errors corresponding to single-nt indels and the remainder corresponding to base substitutions (Quail *et al.* 2012). In cases where there were several hundred CCS reads for a given Can-R mutant, the accompanying background noise was low, especially for base substitutions. To obtain a robust estimate of the base-substitution error frequency across the 2-kb interval sequenced, we combined the CCS reads from hundreds of independent Can-R mutants isolated under low-transcription conditions and determined the error frequency at each position. Two independent SMRT libraries were separately analyzed, and there was an average depth of coverage of ∼15,000 in each. The causative mutation in each independent mutant was excluded from the corresponding CCS reads to uncover background base substitution errors. Subsequently, all the CCS reads from both libraries were then aligned to determine a base-substitution error frequency at each position (Figure 3B). The average error frequency was ∼0.2% across the *CAN1* target sequence with some positions (8/2047) containing no errors; there were only two positions where the error frequency exceeded 1% (1.7% and 1.2% for positions 79 and 91, respectively), and these were adjacent to long homopolymer runs in the 5′ noncoding region. The inset in Figure 3B presents the error frequencies in each SMRT library within a 100-bp interval and reveals highly reproducible values. Although the relative contributions of PCR errors *vs.* SMRT-sequencing errors were not explored, it is possible that use of a higher-fidelity DNA polymerase during amplification would drive the error frequency even lower. With prior knowledge of the error-frequency landscape for a given amplicon, our data indicate that rare variants can be called with very high accuracy.

### Comparing SMRT sequencing to exome sequencing in human lymphomas

To illustrate the broad utility and accuracy of the barcoding-based SMRT sequencing pipeline developed in yeast, we applied the same approach to diffuse large B-cell lymphoma (DLBCL) primary tumors and to DLBCL-derived cell lines. Eleven samples that had been previously characterized by Illumina exome sequencing (Zhang *et al.* 2013) were analyzed by SMRT sequencing that focused on the MYD88 (myeloid differentiation primary response 88; involved in immune responses) and EZH2 (enhancer of zeste homolog 2; a histone methyl transferase) genes. For each sample, three 2-kb genomic segments were individually amplified using barcoded forward–reverse primer pairs: exons 1–4 of MYD88 (with associated introns), exon 5 of MYD88, and exon 16 of EZH2 with flanking intron sequences. The latter two genomic segments harbored mutations identified by exome sequencing and implicated in tumorigenesis. After PCR amplification of the MYD88 and EZH2 segments, the barcoded amplicons were pooled with yeast amplicons for construction of a SMRT library. From 33 barcoded segments subjected to SMRT sequencing, only one failed to return any CCS reads.

A single polymorphism was detected in MYD88 exons 1–4 among the 11 samples analyzed by SMRT sequencing; this polymorphism was in each of the 43 CCS reads for the corresponding sample and hence was homozygous (data not shown). The genetic complexity of the other two genomic segments was high, likely reflecting inherent genetic instability coupled with an unknown tumor-cell ploidy and, for primary lymphoma samples, possible contamination with normal cells. The SNPs present in the CCS data for MYD88 exon 5 and EZH2 exon 16 are summarized in Table 1 and Table S2 and are compared to those previously detected in Illumina exome sequencing. Four and five SNPs were present in the MYD88 and EZH2 amplicons, respectively, used for SMRT sequencing; the reference base at each position is in lowercase and deviations from the reference are in uppercase bold. The number of occurrences of each SNP in the CCS and Illumina reads is given for each sample. The two sequencing methods were in excellent agreement with respect to the homozygous–heterozygous status of SNPs. Only one discrepancy was seen, and this was at position 19 of MYD88. Seventy-nine CCS reads had a C at this position; among Illumina reads, 10 had a C and two had a T.

**Table 1:** Comparison of SMRT sequences and Illumina exome sequences for MYD88 exon 5 (chromosome 3)

DNA Source	Position in MYD88 Exon 5 Amplicon							
	19t		+58g		+1245a		+1524t	
	SMRT	Illumina	SMRT	Illumina	SMRT	Illumina	SMRT	Illumina
Tumor DLBCL773	8t + 65**C**	8t + 22**C**	73g	All g	3a + 70**G**	1**G**	73t	No reads
Tumor DLBCL778	17t + 73**C**	5t + 44**C**	90g	All g	90a	No reads	90t	No reads
Tumor DLBCL799	26t + 29**C**	16t + 21**C**	37g + 18**T**	5g **+** 4**T**	55a	No reads	55t	No reads
Tumor DLBCL816	88t + 19**C**	8t + 3**C**	107g	All g	53a + 54**G**	No reads	107t	No reads
Tumor DLBCL894	165t + 50**C**	27t + 14**C**	215g	All g	315a	No reads	315t	No reads
Tumor DLBCL832	75t	All t	75g	All g	75a	No reads	75t	No reads
Cell line Ly3	79**C**	2t + 10**C**	79g	All g	79**G**	No reads	63t + 16**A**	No reads
Cell line Ly10	16t + 28**C**	7t + 5**C**	44g	All g	16a + 28**G**	No reads	44t	No reads
Cell line SKI	310t	All t	310g	All g	151a + 159**G**	No reads	310t	No reads
Cell line Karpas422	11t	All t	11g	All g	11a	No reads	11t	2t
Cell line Ly1	121t	All t	121g	All g	121a	No reads	121t	No reads

Bases deviating from the reference are uppercase bold. The variant position within exon 5 is numbered relative to the start of the exon. Variants detected downstream of exon 5 are designated “+” and numbered relative to the end of the exon. No reads on the Illumina platform denotes no sequence mapped to a particular region.

### SNP linkage/phasing in tumor samples

**Figure 4:**
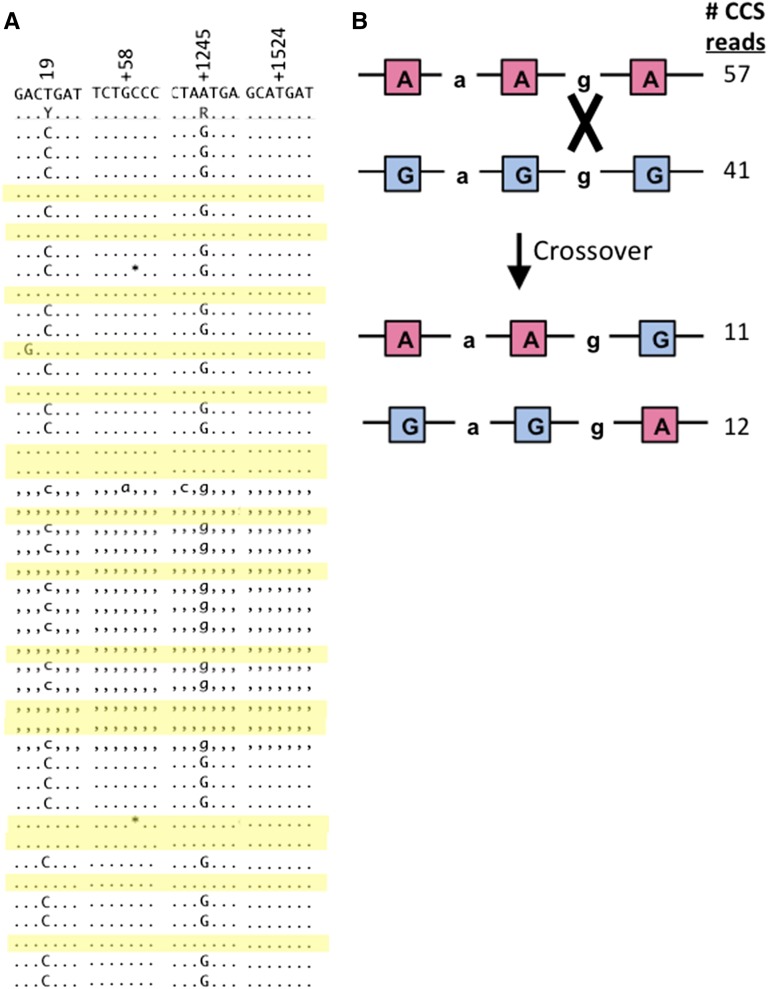
SMRT sequence data from human lymphomas. (A) MYD88 exon 5 CCS reads from tumor DLBCL260. CCS reads with the t-g-a-t haplotype are shaded yellow. (B) Crossover event that results in four haplotypes for the EZH2 exon 16 sequence in tumor DLBCL799.

The distinctive feature of SMRT sequencing relative to other next-gen sequencing methods is read length, which routinely exceeds 10 kb. This greatly extends the length over which SNP linkages can be directly determined and does not require any computational manipulation of the data. Primary tumor DLBCL778, for example, was heterozygous for a single SNP at the first polymorphic position in the MYD88 exon 5 amplicon. Accordingly, 73 CCS reads had haplotype C-g-a-t for and 17 CCS reads had haplotype t-g-a-t; the aligned CCS reads are shown in Figure 4A. Table 2 compiles the SMRT sequencing data for all DLBCL samples and groups the CCS reads by haplotype. The phasing ability of SMRT sequencing is particularly striking in cases in which a polymorphism was present at more than one position. Cell line Ly10 provides one such example, where there was heterozygosity at the first and third SNPs in the MYD88 amplicon, corresponding to four possible haplotypes: C-g-a-t, t-g-a-t, C-g-G-t, and t-g-G-t. SMRT sequencing revealed which two haplotypes were actually present; 28 and 16 CCS reads were C-g-G-t and t-g-a-t, respectively. The phasing revealed in the single-molecule reads was particularly interesting for EZH2 exon 16 in DBLCL799, where three of five SNP sites were heterozygous and there were eight possible haplotypes. There were four distinct haplotypes in the CCS reads, the most abundant of which were G-a-G-g-G and a-a-a-g-a with 41 and 57 CCS reads, respectively. There were two minority haplotypes that were also of similar frequencies: 11 and 12 CCS reads of a-a-a-g-G and G-a-G-g-a, respectively. As illustrated in Figure 4B, the latter two haplotypes can be related to the former two by a reciprocal crossover between the third and fifth polymorphic positions.

**Table 2:** Summary of CCS reads from lymphoma cell lines

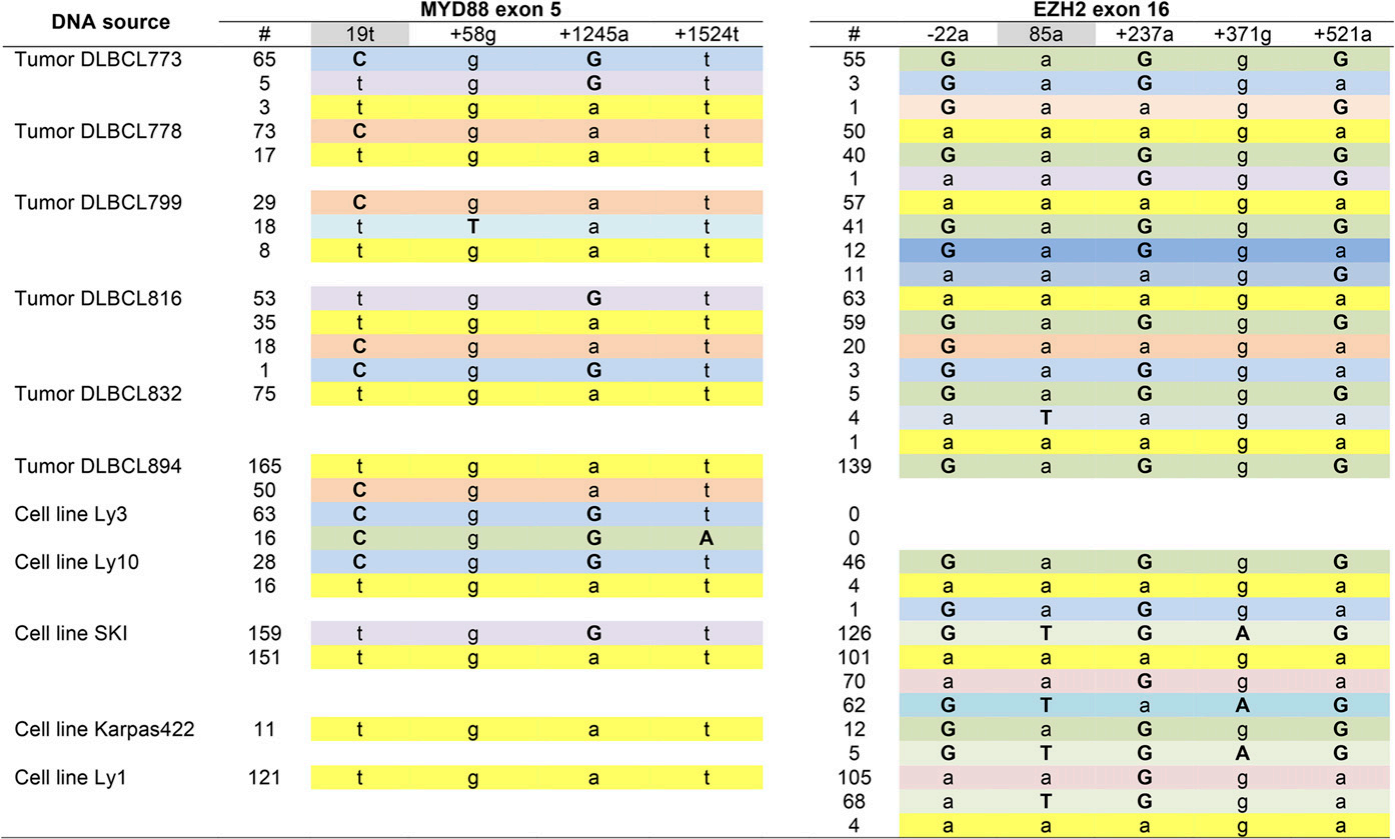

Bases deviating from the reference are uppercase bold, and the site of the causative mutation in each exon is highlighted gray. Haplotypes are color-coded. A variant within a targeted exon is assigned a number based on its position relative to the start of the exon. Noncoding upstream and downstream variant positions are designated − and +, respectively.

## Discussion

In the current study, we document the application of SMRT sequencing as a high-throughput alternative to Sanger sequencing for the analysis of a small number of targets in a large number of independent samples. The key elements in the approach are the unique barcoding of each target during PCR amplification and the pooling of all barcoded amplicons prior to construction of a single SMRT library. Library size is constrained only by available barcodes; with the most recent barcodes developed by PacBio (https://pacbio.secure.force.com/Share/Protocol?id=a1q70000000J4m5AAC), the theoretical limit of unique barcode combinations exceeds 100,000. Following library sequencing, CCS reads are sorted by barcodes and aligned to the appropriate reference sequence. The final step can be done using an automated program that can be downloaded from https://sourceforge.net/projects/smrtseqtool/.

We initially developed the barcoding SMRT-sequencing framework to analyze mitotic recombination intermediates and mutation spectra in budding yeast, where it offers several advantages to Sanger sequencing. First, 2-kb amplicons were sequenced in a single reaction/run, eliminating the need for subsequent assembly of the sequence. With the current average read length of ∼15 kb, it should be possible to efficiently sequence amplicons in the 5-kb range. Second, the unique barcoding of each recombinant/mutant allowed hundreds to be sequenced in one or a few SMRT cells, which greatly reduces the overall sequencing cost. Depending on the specific application, the depth of coverage required will vary and will be the major determinant of how many samples can be sequenced in a single SMRT cell. For mutation identification in yeast, our data suggest that 5–10 CCS reads is sufficient; with ∼50,000 CCS reads for a 2-kb amplicon per SMRT cell, it should be possible to simultaneously sequence several thousand independent mutants. For yeast recombination studies or for analyses of more complex genomes, however, much deeper coverage is required. In our analyses of mutation spectra within a defined yeast gene, we combined ∼30,000 CCS reads obtained using two independent libraries to measure the base-substitution error frequency at each position across the 2-kb amplicon. The average error frequency was only 0.2%, but it varied from position to position. Between libraries, however, the error frequencies at independent positions were in excellent agreement. These data suggest that, with prior knowledge of the error profile, rare variants in a more complex sample can be identified with high accuracy. Moreover, as we demonstrate, a real mutation occurred in virtually every read. Thus, one needs to only look at the aligned reads provided to identify the causative mutation; no statistical analysis is needed.

The barcoding-SMRT sequencing pipeline was developed in yeast, but its broader utility was demonstrated by applying the same approach to independent lymphoma samples. We analyzed 2-kb segments within the MYD88 and EZH2 genes and validated the specific changes detected using prior Illumina exome-sequencing data. Importantly, SMRT sequencing readily revealed the linkage relationships between polymorphic SNPs and the complexity of the haplotypes present in the samples analyzed. Although linkage relationships can be determined using other next-gen methods, it requires sophisticated computational methods (Snyder *et al.* 2015). As an alternative to whole genome or exome sequencing of tumor samples, it should be straightforward to barcode multiple independent targets of interest within a single genome or within multiple genomes. This may be particularly useful in screening tumors for associated defects in DNA repair pathways, which can inform subsequent chemotherapy, or for identifying mutations in genes whose products are potentially drugable. With a high depth of coverage and prior knowledge of the error landscape, it also should be possible to identify rare variants with high confidence.

The current study focused primarily on high-throughput identification of mutations in specific targets, but the general framework can be readily adapted to other types of analyses. The combined barcoding, SMRT-sequencing approach, for example, is particularly well suited for characterization of splice variants in different tissues or genotypes. Different CCS reads will directly reflect splice variants, and their relative proportions should reflect the abundances of the corresponding mRNAs (Sharon *et al.* 2013). Other potential applications include examination of multiple markers for fungal species characterization (Schoch *et al.* 2012), validation of kilobase-sized oligonucleotides in synthetic systems (Gibson *et al.* 2010), rapid screening of various DNA-binding substrates for engineered proteins (Thyme *et al.* 2014), and examining genome alterations made by engineered endonucleases (Hendel *et al.* 2014). As the technology improves, especially with regard to read length by single DNA polymerases and error reduction, applications for SMRT technology will continue to evolve.

## Supplementary Material

Supporting Information
