# Supplementary material for: SMRT Sequencing for Parallel Analysis of Multiple Targets and Accurate SNP Phasing
Source: G3 (Bethesda). 2015 Oct 22;5(12):2801–8. doi: 10.1534/g3.115.023317 (PMC4683651; doi:10.1534/g3.115.023317)
Supplement: Supporting Information [file supp_5_12_2801__index.html]

SMRT Sequencing for Parallel Analysis of Multiple Targets and Accurate SNP Phasing — SMRT Sequencing for Parallel Analysis of Multiple Targets and Accurate SNP Phasing — Supporting Information 

# SMRT Sequencing for Parallel Analysis of Multiple Targets and Accurate SNP Phasing

## Supporting Information for Guo *et al.*, 2015

**Files in this Data Supplement:**

- Figure S1 - Sequence alignment for variant calling. (A) base substitution (B) 40-bp deletion (C) complex events. (.pdf, 87 KB)
- Table S1 - Forward and reverse barcode sequences used in SMRT sequencing. (.docx, 18 KB)
- Table S2 - Comparison of SMRT sequences and Illumina exome sequences for EZH2 exon 16 (chromosome 7). (docx, 118 KB)
