# Supplementary material for: SMRT Sequencing for Parallel Analysis of Multiple Targets and Accurate SNP Phasing
Source: G3 (Bethesda). 2015 Oct 22;5(12):2801–8. doi: 10.1534/g3.115.023317 (PMC4683651; doi:10.1534/g3.115.023317)
Supplement: Supporting Information [file supp_g3.115.023317_FigureS1.pdf]

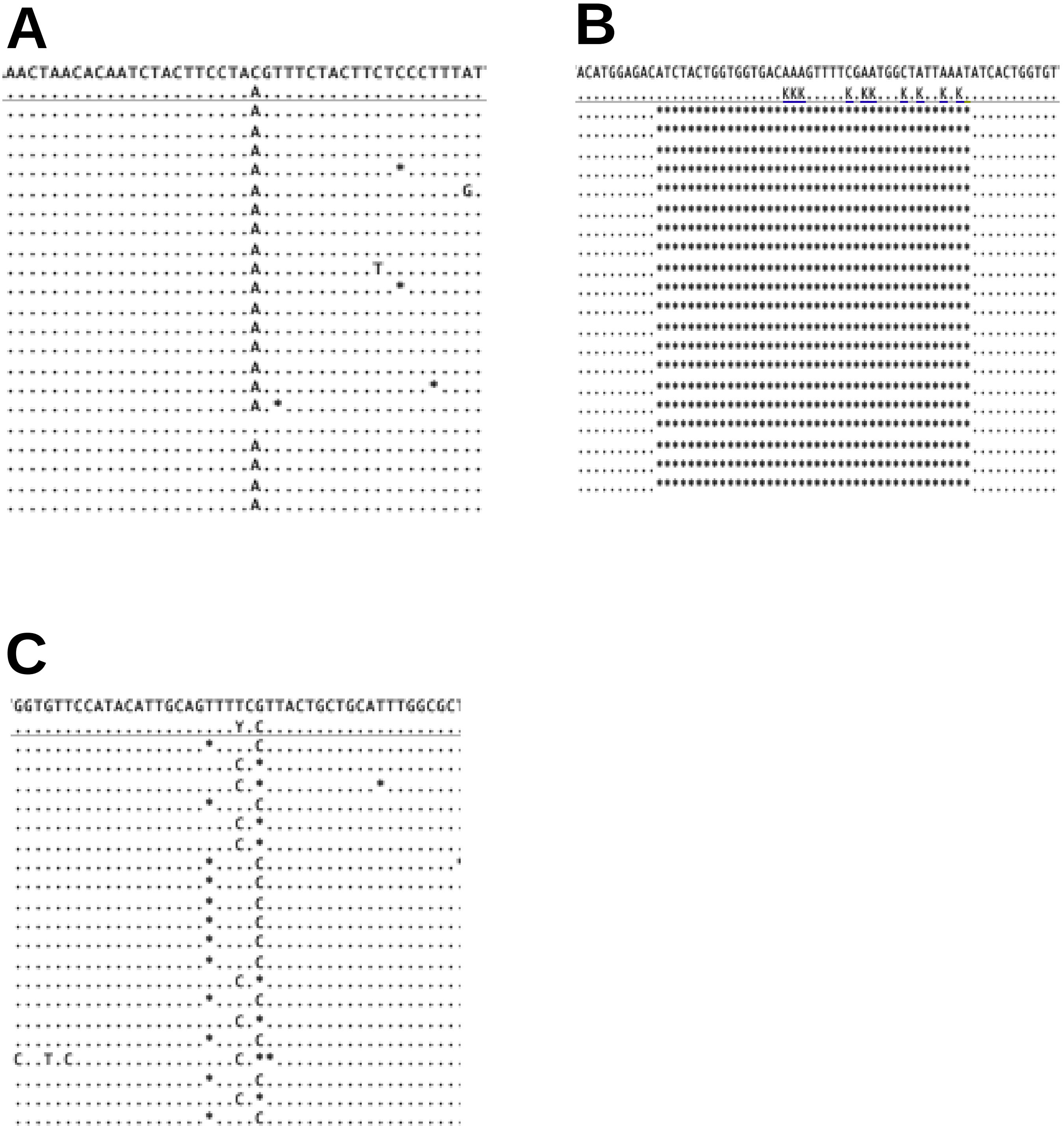

Figure S1. Sequence alignment for variant calling. (A) base substitution (B) 40-bp deletion (C) complex events.
