# Supplementary material for: SMRT Sequencing for Parallel Analysis of Multiple Targets and Accurate SNP Phasing
Source: G3 (Bethesda). 2015 Oct 22;5(12):2801–8. doi: 10.1534/g3.115.023317 (PMC4683651; doi:10.1534/g3.115.023317)
Supplement: Supporting Information [file supp_g3.115.023317_Table_S1.docx]

**Table S1. Forward and reverse barcode sequences used in SMRT sequencing**

| Forward barcodes | 5’ to 3’ sequence |
| --- | --- |
| F1 | GCGCTCTGTGTGCAGC |
| F2 | TCATGAGTCGACACTA |
| F3 | TATCTATCGTATACGC |
| F4 | ATCACACTGCATCTGA |
| F5 | ACGTACGCTCGTCATA |
| F6 | TGTGAGTCAGTACGCG |
| F7 | AGAGACACGATACTCA |
| F8 | CTGCTAGAGTCTACAG |
| F9 | AGCACTCGCGTCAGTG |
| F10 | TCATGCACGTCTCGCT |
| F11 | AGAGCATCTCTGTACT |
| F12 | CGCATCGACTACGCTA |
| F13 | CGTAGCGTGCTATCAC |
| F14 | ATGCTGATGACTGCGA |
| F15 | TGCGTGAGCTGTACAT |
| F16 | CGATCATCTATAGACA |
| F17 | CGACGTATCTGACAGT |
| F18 | CACGTCACTAGAGCGA |
| F19 | TGTCGCAGCTACTAGT |
| F20 | CATACGCTGTGTAGCA |
| F21 | AGTCGCATGACTGTGT |
| F22 | CAGTACTGCACGATCG |
| F23 | GTGCTGAGCATCAGAC |
| F24 | CACTGATCGATATGCA |
| F25 | TACAGTGTCTGCTGCG |
| F26 | TACAGATAGTGTAGCG |
| F27 | TCGTAGAGCTCGAGAC |
| F28 | GAGCTGCGCACTCGAT |
| F29 | GCGATGTCGCTATGTG |
| F30 | CGAGAGTCAGCGCATA |
| F31 | TCACGATGAGCACGTA |
| F32 | GACTGAGATCATGATC |
| F33 | ACGACATGATACTGCT |
| F34 | ATACAGCACAGATGTG |
| F35 | ACAGTCGATATCTCTC |
| F36 | GCTCGATCACATGACG |

| Reverse barcodes | 5’ to 3’ sequence |
| --- | --- |
| R1 | TCATATGTAGTACTCT |
| R2 | GCGATCTATGCACACG |
| R3 | TGCAGTCGAGATACAT |
| R4 | GACTCTGCGTCGAGTC |
| R5 | TACAGCGACGTCATCG |
| R6 | GCGCAGACTACGTGTG |
| R7 | GTCTCTGCGATACAGC |
| R8 | AGTATGAGATAGCTCG |
| R9 | GCGACGAGTACTCATG |
| R10 | AGTATCACAGTCGCTG |
| R11 | ATCATATGATGCGACA |
| R12 | AGACGTAGATCACAGC |
| R13 | CGTGTCATGCTACTCA |
| R14 | TGTGAGACTGCATGTC |
| R15 | GCTCAGTGCGCTACTG |
| R16 | ACTATCGCGCACGCAG |
| R25 | TCAGCTGACGATGTGA |
| R26 | ACTGATGCGCACATGT |
| R27 | CTACTCTCAGCAGTGA |
| R28 | ATCTACATCACGACTC |
| R29 | ATATAGTACAGCGTCT |
| R30 | GACACGACTAGATCGC |
| R31 | TACGAGTCTGTCATAC |
| R32 | ACTCAGCTACATAGTG |
| R33 | ACGTATCATAGTGAGA |
| R34 | GAGTCGTATCGCTCAT |
| R35 | GCGATCACGAGTAGAC |
| R36 | CTAGACGTACATGTCG |
| R37 | TAGCAGTCACTGTGCG |
| R38 | CGTCATGCGATAGCTA |
| R39 | GCGCAGTCGTCTGTAT |
| R40 | ATGAGCTACGTACAGA |
| R41 | GTCGCGAGTCTATCAG |
| R42 | ACATCGATCTGCACTA |
| R43 | AGTATAGCATAGACGC |
| R44 | GTGAGAGCGTGACTCT |
| R45 | TGTCAGTAGATGACTC |
| R46 | TCGTACGAGATCGACA |
| R47 | CTACATGTGACTCGAG |
| R48 | GCGCTATAGTGCTCGT |
