# Supplementary material for: SMRT Sequencing for Parallel Analysis of Multiple Targets and Accurate SNP Phasing
Source: G3 (Bethesda). 2015 Oct 22;5(12):2801–8. doi: 10.1534/g3.115.023317 (PMC4683651; doi:10.1534/g3.115.023317)
Supplement: Supporting Information [file supp_g3.115.023317_Table_S2_.docx]

**Table S2. Comparison of SMRT sequences and Illumina exome sequences for EZH2 exon 16 (chromosome 7)**

| DNA source | Position in EZH2 exon 16 amplicon | | | | | | | | | |
| --- | --- | --- | --- | --- | --- | --- | --- | --- | --- | --- |
|  | -22a | | 85a | | +237a | | +371g | | +521a | |
|  | SMRT | Illumina | SMRT | Illumina | SMRT | Illumina | SMRT | Illumina | SMRT | Illumina |
| Tumor DLBCL773 | 59**G** | All **G** | 59a | All a | 1a+58**G** | no reads | 59g | no reads | 3a+56**G** | no reads |
| Tumor DLBCL778 | 51a+40**G** | a + **G** | 91a | All a | 50a+41**G** | no reads | 91g | 2g | 50a+41**G** | 3a |
| Tumor DLBCL799 | 68a+53**G** | 11a+4**G** | 121a | All a | 68a+53**G** | 1a | 121g | no reads | 69a+52**G** | no reads |
| Tumor DLBCL816 | 63a+82**G** | 3a+3**G** | 145a | All a | 83a+62**G** | no reads | 145g | no reads | 86a+59**G** | no reads |
| Tumor DLBCL894 | 139**G** | All **G** | 139a | All a | 139**G** | 1**G** | 139g | 1g | 139**G** | 1**G** |
| Tumor DLBCL832 | 5a+5**G** | 11a+8**G** | 6a+4**G** | 22a+5**T** | 5a+5**G** | no reads | 10g | no reads | 5a+5**G** | no reads |
| Cell line Ly3 | No reads | 1a+4**G** | No reads | All a | No reads | no reads | No reads | no reads | No reads | no reads |
| Cell line Ly10 | 4a+47**G** | 2a+2**G** | 51a | All a | 4a+47**G** | no reads | 51g | no reads | 5a+46**G** | no reads |
| Cell line SKI | 171a+188**G** | 4a+6**G** | 171a+188**T** | 4a+10**T** | 163a+196**G** | 1a+2**G** | 171g+188**A** | 1g+1**A** | 171a+188**G** | 2a |
| Cell line Karpas422 | 17**G** | 4**G** | 12a+5**T** | 3a+3**T** | 17**G** | no reads | 12g+5**A** | no reads | 17**G** | no reads |
| Cell line Ly1 | 177a | All a | 109a+68**T** | 19a+10**T** | 4a+173**G** | 1**G** | 177g | 1g | 177a | 3a |

See Table 1 for description.
